# The concept, dimensions, and measurement of innovative competitive behavior of Chinese scientific and technological personnel

**DOI:** 10.3389/fpsyg.2025.1449998

**Published:** 2025-11-19

**Authors:** Ping Li, Weihua Fang, Guannan Hou, Rui Li

**Affiliations:** 1 School of Public Management, Inner Mongolia University, Hohhot, China; 2 School of Public Administration, Beihang University, Beijing, China; 3 Institutes of Science and Development, Chinese Academy of Sciences, Beijing, China

**Keywords:** innovative competitive behavior, innovation, competition, grounded theory, scale development

## Abstract

**Introduction:**

While competitive behavior in innovation activities is increasingly prevalent in practice and actively promoted by policymakers, it remains an under explored area in academic research. To bridge this gap, we introduce the concept of “innovative competitive behavior,” providing a conceptual definition and clarifying its theoretical boundaries, rooted in social comparison theory from psychology.

**Methods:**

A mixed-methods approach was employed to systematically examine this construct. First, a qualitative study guided by grounded theory was conducted to explore the core dimensions of the construct. Subsequently, a quantitative phase involved the development and validation of a psychological scale through questionnaire surveys and factor analysis.

**Results:**

The qualitative phase identified two core dimensions: competitive behavior in innovation input (including acquiring, allocating, and utilizing resources) and competitive behavior in innovation output (involving competition in the quantity, quality, and outcomes of innovation). These dimensions form the foundation of the proposed Four-Degree Diamond Model of innovative competitive behavior. In the quantitative phase, a 13-item Innovative Competitive Behavior Scale was developed and validated, demonstrating satisfactory reliability and validity.

**Discussion:**

By integrating innovation theory and competition theory at the micro level, this research contributes to the emerging literature on individual-level innovative competition and establishes a theoretical foundation for future studies. These findings offer a roadmap for policymakers and corporate managers to effectively incentivize scientific and technological personnel, thereby enhancing innovation performance through innovative competition.

## 1 Introduction

Innovation entails not only collaboration but also competition among scientists and engineers (Anderson et al., 2007). Competition is increasingly recognized as a significant driving force behind the development of science and technology (Rathi, 2014). To harness its potential, policymakers, including those in China, are actively implementing competitive mechanisms in research management. Notable recent examples are the “Enlisting and Leading” and “Horse Racing” initiatives (Zeng and Huang, 2023; Sun, 2022). The “Enlisting and Leading” model fosters open competition, selecting solvers for specific challenges based on merit regardless of seniority (Zeng and Huang, 2023). Conversely, the “Horse Racing” approach encourages ongoing competition among diverse research groups to achieve project milestones (Sun, 2022). These competitive mechanisms aim to stimulate innovation by intensifying competitive pressures, affecting not only institutions and teams but also individual scientists and technologists within them (Chen et al., 2022).

This environment places individual researchers in contexts where recognition, resource allocation, and career progression are often tied to competitive outcomes. While substantial research examines innovation competition at the organizational level (e.g., among firms) (Hong, 2024; Zhang, 2024) and focuses on structured innovation contests or government-led competitions (Bai, 2022; Wooten, 2022; Dargahi et al., 2021), a critical gap remains. The everyday, psychologically driven innovative competitive behavior (ICB) exhibited by individual scientists and technologists striving for advantage within their professional sphere is poorly conceptualized and lacks a validated measurement tool. Existing scales measure either innovative behavior or competitive behavior in isolation, but none capture the specific construct of individual-level ICB integrating both aspects. Studies on academic competition often address broader structural or motivational factors (Matusof, 2024) rather than the specific behavioral dimensions of competition in innovation contexts.

Conceptually, ICB can be understood through the lens of social comparison theory (Festinger, 1954), where individuals evaluate their abilities and achievements relative to peers, potentially motivating competitive actions aimed at gaining an edge in innovative pursuits. To address this research gap, this study employs an exploratory sequential mixed-methods approach. First, in-depth qualitative interviews were conducted with scientific and technological personnel to explore their experiences and perceptions. Using the grounded theory approach, the core dimensions and specific manifestations of the information cocoon effect were identified. Based on these insights, we subsequently developed and validated a set of scales through questionnaire surveys and factor analysis, which were specifically designed to measure an individual’s innovative competitive behavior (ICB). The primary objectives of this research are to:

1. Conceptualize the multi-dimensional construct of individual Innovative Competitive Behavior (ICB) based on empirical evidence and grounded in social comparison theory.
2. Develop and validate a reliable and valid measurement scale for ICB.
3. Explore the potential relationships between ICB dimensions and factors such as innovation input and output.

Grounded theory is a qualitative research method developed by Glaser and Strauss (1967) that emphasizes extracting theory from empirical data through systematic operations and practical observation while constructing theory from the bottom up. The decision to use grounded theory for developing the dimensions of innovative competitive behavior among scientific and technological personnel in this paper is based on the following considerations. First, research on competitive behavior in innovative activities within academia is lacking, highlighting the need to explore its types. Grounded theory is particularly suitable for investigating new topics. Second, the competitive behavior of scientific and technological personnel in innovation activities is complex, and grounded theory excels at revealing and explaining social processes and intricate patterns, which aids in refining and analyzing its structure. Third, this behavior is widespread in practice, facilitating data collection, and grounded theory is well equipped to handle large volumes of textual material. The development of the scale needs to be based on existing research while also considering the meaning of the concept itself. With reference to the literature by Howell et al. (2020), we used a combination of qualitative and quantitative research methods to develop a scale for the concept of innovative competitive behavior. We first use grounded theory to construct the dimensions of innovative competitive behavior, ensuring that it comprehensively reflects the connotation of the concept. Based on these dimensions of innovative competitive behavior, and by referencing existing scales, we can design several measurement items under each dimension. At this moment, we obtained the initial scale of innovative competitive behavior. This study conducts a questionnaire survey to gather data and undertakes a preliminary investigation on the initial scale. After some items are eliminated and modified, the final scale for innovative competitive behavior is established. The reliability and validity of the final scale must be tested to ensure its effectiveness. Thus, another questionnaire survey is conducted to collect data for formal research, utilizing methods such as Cronbach’s alpha coefficient, exploratory factor analysis, and confirmatory factor analysis to evaluate the scale’s quality.

Understanding individual ICB is crucial. Scientists and technologists are fundamental agents of knowledge creation (Iazzolino and Laise, 2016, Iazzolino et al., 2017; Iazzolino and Laise, 2018), and their behavioral responses to competition directly impact innovation outcomes. This research aims to contribute by: (1) providing a conceptual framework and validated tool for measuring ICB, filling a key methodological gap; (2) enhancing understanding of how competition manifests behaviorally at the individual level in innovation settings; and (3) extending the application of social comparison theory to the domain of individual competition in scientific and technological innovation. Practically, insights into ICB can inform strategies for research management, talent development, and fostering healthier competitive environments conducive to sustainable innovation.

## 2 Literature review

### 2.1 Research on innovation competition

At present, the relevant literature on “innovative competitive behavior” is still relatively limited, and it can mainly be classified into two categories: innovation contest and academic competition.

Innovation contests are significant manifestations of individual competition in innovation activities. These contests are related to innovation activities and are implemented mainly through procedures such as defining problems, setting awards, selecting participants, determining processes, and building platforms (MacCormack et al., 2013). It has been argued that innovation competitions serve several purposes: First, these competitions foster competition in open innovation activities, motivating many potential innovators and various organizations to engage in innovation. The diverse participants, who come from different backgrounds and viewpoints, can generate a plethora of solutions and breakthrough ideas. Second, innovation competitions offer participants opportunities to sharpen their skills and network with others. Finally, these competitions can advance research in unfamiliar fields and problems while identifying and evaluating early opportunities (MacCormack et al., 2013). Schweitzer et al. (2012) indicated that idea competitions can provide essential inputs for decision-making in the early stages of product innovation, yielding more and better ideas at a lower cost. MacCormack et al. (2013) noted that innovation contests also require attention to several issues. First, the benefits of using competition to pursue innovation must be weighed against potential costs and risks. Second, while material rewards can incentivize potential participants, the focus should not be solely on these rewards. Third, managing and operating innovation competitions incur high costs. Fourth, the outcomes of innovation competitions carry disclosure risks. Fifth, these competitions mean that organizers relinquish some control to the participants. Sixth, innovation competition may lead to resource duplication (MacCormack et al., 2013).

In addition to innovation contests, scientists also engage in academic competition, representing a form of competitive behavior in innovation activities. Matusof (2024) found that other-referent competition in academic environments is positively correlated with bullying behavior, whereas task-oriented competition is moderately negatively correlated with bullying behavior. “Academic involution” is also characterized by irrational competition among researchers regarding paper writing and publication (Wang, 2024). This state essentially represents a type of competition in innovative activities. Wang et al. (2024) noted that “involution” has become a buzzword in recent years within the field of Chinese higher education, leading to heightened competition and anxiety. Liu et al. (2024) found that the atmosphere of academic involution can impact college students’ stress responses.

Innovative competitions and academic competitions highlight the genuine presence of individual innovation competitions. However, there is a lack of clear understanding of this phenomenon in academia, and it has rarely been explored. We utilize CiteSpace 6.1 software to organize and validate further research efforts related to innovation competition.

**FIGURE 1:**
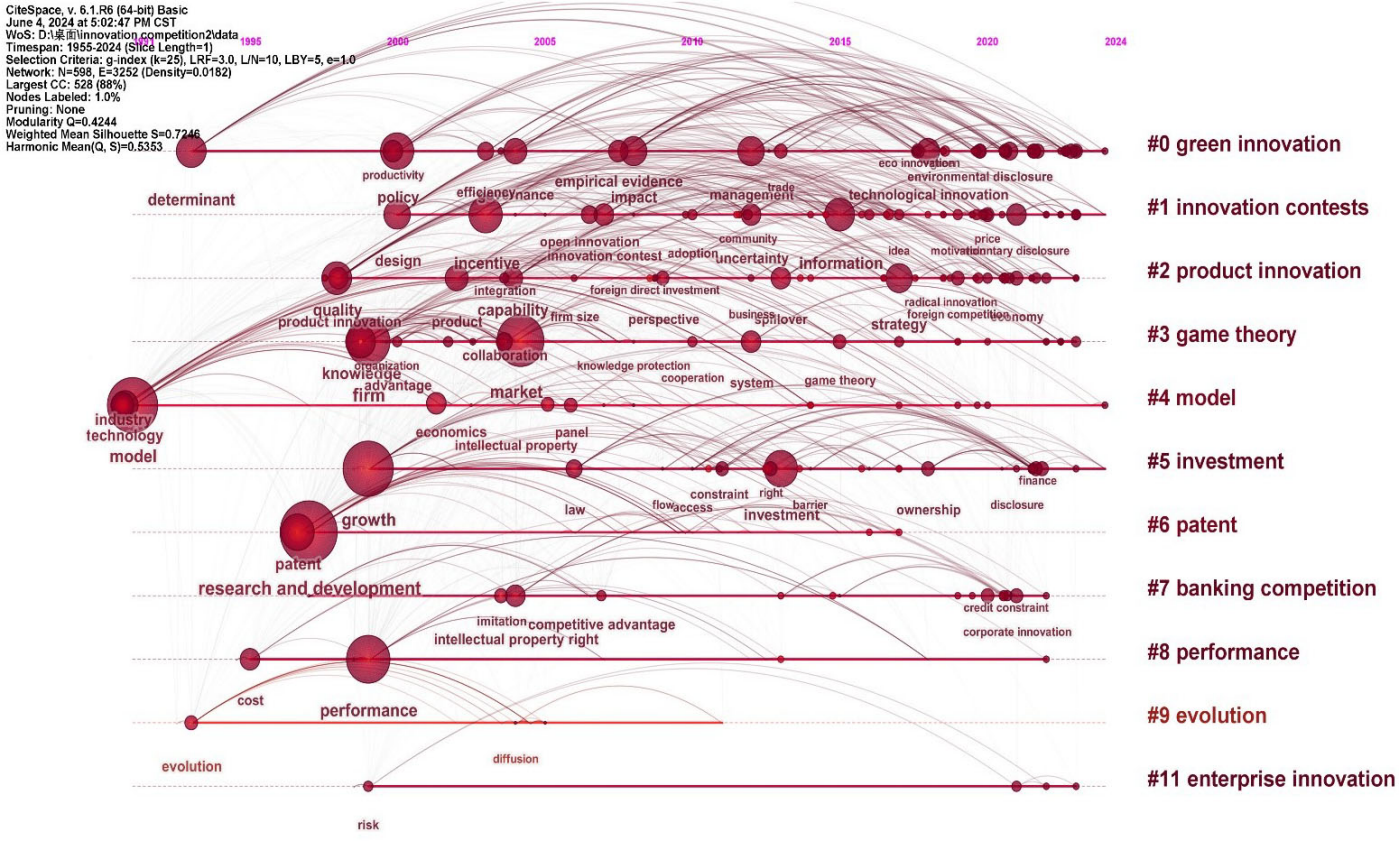
A timeline of keyword evolution in the field of international innovation competition research (1955–2024).

Within the Web of Science (WoS) SCI and SSCI databases, a thorough search was conducted using keywords such as “innovation contest,” “innovative contest,” “innovation competition,” “innovative competition,” “innovative race,” “innovation race,” “innovation disclosure,” and “innovation protection.” The search timeframe was set from 1912 to June 1, 2024. After careful filtering to exclude irrelevant or invalid documents, a refined collection of 1078 pertinent search records was compiled. Notably, terms such as “patent race,” “research and development competition,” and “property protection,” which allude to facets of innovative competition, were deliberately excluded from the search criteria to avoid introducing bias and ensure an impartial portrayal of the evolving trends in innovative competition research. Thus, our search was narrowed to encompass only terms closely linked to “innovative competition.” The specific results are shown in Figure 1.

The timeline chart of research on innovation competition reveals that the academic community has engaged in innovation competition across various fields, such as enterprises, banks, investment, patents, products, and environmental protection. However, within the context of individual-centered innovation competition, the primary focus of the research revolves around innovation contests, with a notable absence in examining individual innovative competitive behavior. By further tracing the evolution of the integration of innovation and competition within the academic landscape, we observe that the theory of corporate innovation competition has flourished, accompanied by a substantial and growing body of research. In contrast, innovative competition at the governmental and individual levels remains in its nascent phase, characterized by a relatively insufficient research foundation. Therefore, it is essential to focus on the individual level.

### 2.2 Research on innovative behavior and competitive behavior

By reviewing relevant studies on innovative and competitive behavior, we aim to explore the research potential of individual innovative competitive behavior. While research on innovative behavior and competitive behavior at the personal level is abundant, studies on individual-level innovative competition remain limited. Researchers have engaged in numerous discussions regarding the conceptual definitions, measurement methods, and influencing factors of innovative and competitive behavior. Scott and Bruce (1994) believed that innovative behavior consists of multiple stages, including personal problem recognition, idea formation, seeking help and support for creative ideas, experimentation and practice with innovative ideas, and ultimately creating commercial products or services. Other scholars argue that innovation behavior includes not only the innovation idea itself but also the generation of the idea, its content, the promotion of the idea, and the implementation plan. All of these elements are necessary conditions to ensure the effective realization of the innovation idea (Zhou and George, 2001). From a process perspective, this paper also concurs that innovative behavior entails the generation, evaluation, execution, promotion, and dissemination of innovative ideas.

The theoretical foundation of both competitive behavior and innovative competitive behavior is social comparison theory in psychology. According to this theory, social comparison involves comparing one’s abilities, feelings, and circumstances with those of others. The existing research suggests that there are three primary types of social comparison: lateral comparison, upward comparison, and downward comparison. Lateral comparison refers to the tendency of individuals to compare themselves with others who have similar abilities and viewpoints (Festinger, 1954). Upward comparison indicates the tendency of individuals to compare themselves with others of higher status to identify gaps and achieve personal improvement (Suls and Wheeler, 2013). Downward comparison relates to the tendency of individuals experiencing negative emotions to compare themselves with others who are worse off, thereby enhancing their own subjective wellbeing (Wills, 1981). The core meaning of competition is comparison. Competition is an event in which at least two parties participate (Tsai, 2002). In a competitive context, interpersonal competition can be defined as a situation where there is a negative correlation between participants’ goals (Deutsch, 1949). In general, only one party in a competition can successfully achieve its goal. Therefore, competition can be defined as the efforts of an individual or group to achieve a zero-sum outcome (Swab and Johnson, 2019). From the perspective of competitive tendency, some scholars define individual competitive ability as the willingness to participate in interpersonal competition and the desire to perform better than others do (Spence and Helmreich, 1983). This essentially contains the implications of comparison. Another perspective combines competitive situations with competitive tendencies, arguing that competition is a contest between individuals (or collectives or nations). Competition arises when two or more parties strive to obtain something that is not available to all parties (Eatwell et al., 1988). Competitive behavior refers to the tendency of individuals to adopt confrontational methods in interpersonal interactions to gain benefits for themselves (Swab and Johnson, 2019). This type of confrontation also implies comparison with others.

At present, the scales for measuring innovation behavior are quite mature. Some examples of widely recognized, representative, and frequently used scales include the six-item scale by Scott and Bruce (1994), the nine-item scale by Janssen (2000), and the thirteen-item scale by Zhou and George (2001). Currently, many scholars have measured individual competitive behavior by examining competitive intentions. As proposed by Smither and Houston (1992), the individual competitive index and its subsequent modified version measure an individual’s general sense of competition, primarily emphasizing the competitive spirit that individuals display regardless of time or place. Tost et al. (2012) subsequently evaluated the competitiveness of the subjects by asking them to complete the revised competitiveness index scale. In addition, there are scales that measure individual competitive behavior according to specific situations. Among them, the three-item scale developed by Hays and Bendersky (2015) is widely used.

**FIGURE 2:**
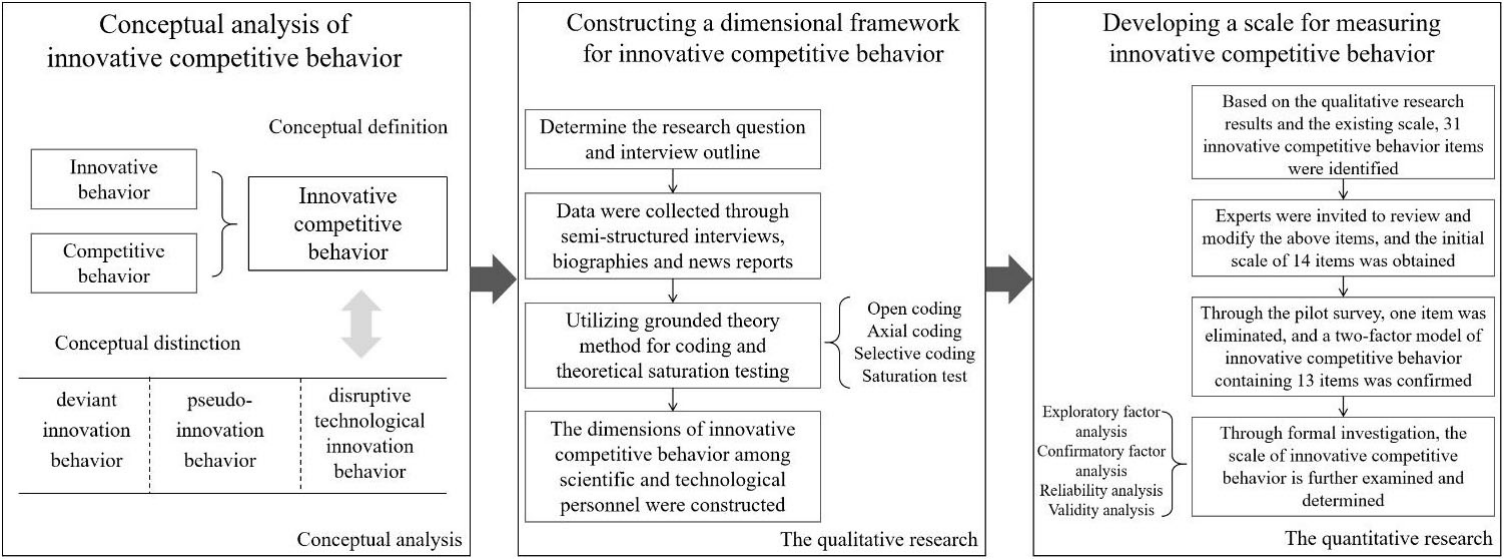
Research roadmap.

In conclusion, the existing research has not yet defined the scope of “innovative competition.” Furthermore, studies on innovative competition have focused primarily on the organizational level. Individual participation in an innovation contest essentially falls within the realm of individual innovative competition. However, despite the relative maturity of social comparison theory and the early introduction of the concept of competitive behavior, the notion of individual innovation competitive behavior has yet to emerge, along with its relevant dimensions and psychological scales. Individual “competition for innovation activities” is widespread globally and is strongly advocated by the government. In reality, the issue of innovation involution is becoming increasingly profound and prominent. To better develop scientific and technological human resources, it is necessary to conduct more in-depth academic discussions on these topics. Innovative competitive behavior represents a pivotal factor in innovative performance. The study of individual innovative competitive behavior cannot only explore effective ways to enhance innovation performance but also integrate innovation and competition at the micro-individual level, thereby opening new avenues for research on individual innovative competition and enriching the theory of innovation, competition theory, and the theoretical system of innovation competition. Furthermore, innovative competitive behavior originates from social comparison theory in psychology, and our research expands the practical application scope of social comparison theory. Consequently, this paper is organized as follows: First, by combining the concepts of innovative behavior and competitive behavior, we propose the concept of “innovative competitive behavior” and define its scope. Second, the grounded theory method is adopted to establish the dimensions of innovative competitive behavior. Finally, utilizing methods such as questionnaire surveys and factor analysis, we validate these dimensions and establish the Innovative Competitive Behavior Scale. Please refer to Figure 2 for further details.

### 2.3 Defining the concept of innovative competitive behavior

**FIGURE 3:**
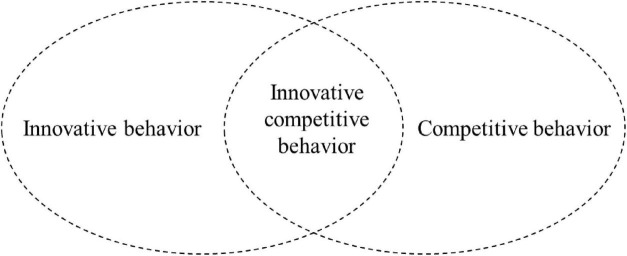
Venn diagram of the concept of innovative competitive behavior.

Innovation competition exists among teams, enterprises, governments, and nations. This study begins from an individual perspective and focuses primarily on innovative competitive behavior at the individual level. We designate the phenomenon of “competition within innovative activities” as “individual innovative competitive behavior.” Before offering a clear conceptual definition, we first discuss the scope of the concept. “Competition in innovative activities” implies that innovative competitive behavior is a subset of innovative behavior, further suggesting that innovative competitive behavior consists of competitive conduct within the realm of innovation activities without extending beyond them. Therefore, innovative competitive behavior is also a subset of competitive behavior. Consequently, innovative competitive behavior represents the intersection of innovative behavior and competitive behavior (Figure 3), indicating competitive behavior in innovation and innovative behavior in competition.

Innovative competition at the individual level and competitive contests encouraged by the government are not merely competitive behaviors; they represent competitive actions pursued to achieve innovation objectives. When the types of behaviors involved are considered, competitive behavior includes not only innovative competitive behavior but also imitative competitive behavior. Similarly, innovative behavior encompasses not only innovative competitive behavior but also collaborative behaviors aimed at fostering innovation. Thus, at the intersection of these two behaviors, the concept of innovative competitive behavior is sound.

Innovative behavior encompasses the generation, evaluation, implementation, promotion, and dissemination of innovative ideas (Berman and Kim, 2010). Competition involves a contest between at least two parties, representing a rivalry among individuals (or groups or nations). Whenever two or more parties strive to obtain something that cannot be shared among all, competition exists (Eatwell et al., 1988). By integrating the concepts of innovative behavior and competitive behavior, we define individual-level innovative competitive behavior as the activities individuals engage in to achieve specific goals while competing with others in the generation, evaluation, execution, facilitation, and promotion of new ideas. According to Tsai’s (2002), view competitive behavior possesses selfish and confrontational characteristics. In other words, individuals tend to adopt confrontational methods to address disagreements and seek to secure favorable outcomes in social interactions to gain control and master their current status. As a form of innovative competitive behavior, it also displays selfish and confrontational traits. This is primarily evident in how innovative competitive behavior is often pursued to meet specific objectives, reflecting its selfish nature. Moreover, as innovative competitive behavior occurs in social interactions, it also presents confrontational characteristics. Additionally, innovative competitive behavior is continuously evolving and influenced by various factors, such as individuals and the external environment, and it possesses dynamic traits. While competitive behavior manifests in various groups, innovative behavior is mainly exhibited by scientific and technological personnel. Therefore, at the intersection of competitive and innovative behavior, the primary actors in innovative competitive behavior are scientific and technological personnel. This paper primarily examines the innovative competitive behavior of scientific and technological personnel.

## 3 Dimension construction of innovative competitive behavior—qualitative research

### 3.1 Materials and methods

The grounded theory method was adopted to analyze the “Concepts and Types of Innovative Competitive Behavior among Scientific and Technological Personnel.” Primary data were collected through semi-structured interviews with scientific and technological personnel, whereas secondary data were gathered through a literature review. By engaging in open, axial, and selective coding of the collected texts, we constructed a multifaceted framework outlining the dimensions of innovative competitive behavior among scientific and technological personnel.

The focus of this paper is on scientific and technical personnel. UNESCO defines “scientific and technological personnel” as “all those who are directly engaged in scientific and technological activities in an institution or unit, usually for remuneration, and who comprise the groups of scientists and engineers, technicians and support staff” (UNESCO, 1979). Among the fields of science and technology are natural sciences, engineering, medicine, agriculture, social sciences, and humanities (UNESCO, 1979). Compared with undergraduates, master’s and doctoral students often have their own research directions and undertake specific scientific research tasks. Therefore, we believe that master’s and doctoral students should be classified as scientific and technological personnel. The 29 scientific and technological personnel we interviewed are involved in various industries, including aerospace, intellectual property, education, machinery, agriculture, medicine, and administration. They come from different organizations, such as central enterprises, private enterprises, universities, and scientific research institutes, which serve as suitable representatives. The average length of the interviews was 1 h. The basic information of the interviewees is presented in Table 1.

**TABLE 1:** Basic information about the interviewees.

Number	Participant	Gender	Education attainment	Position	Work content/ research field	Type of organization	Work experience (year)	Interview duration
1	Zhao**	Female	Bachelor	Intellectual property manager	Intellectual property rights	Corporation	4–6	1.5 h
2	Yan**	Male	Master	Agricultural R&D staff	Crop science	Corporation	≥ 10	2 h
3	Liu**	Male	Doctor	University teacher	Urban planning	University	7–9	1.5 h
4	Wang*	Female	Master	Doctoral student	Policy deliberation	University	0	2.5 h
5	Liu*	Female	Bachelor	Technology transfer specialist	Technology transfer	Corporation	1–3	40 min
6	Wei**	Female	Doctor	University teacher	Marine technology	University	7–9	2.5 h
7	Liu**	Female	Master	Doctoral student	Civil and commercial law	University	0	45 min
8	Zhang**	Female	Bachelor	Chemical process technician	Chemical engineering and technology	Corporation	4–6	35 min
9	Nie**	Male	Master	Doctoral student	Aerospace	University	0	3.5 h
10	Gao*	Male	Master	Engineer	Weapons research	Corporation	1–3	2 h
11	Liu**	Female	Bachelor	Civil servant	Network management	Government	1–3	40 min
12	Yao*	Male	Bachelor	Agricultural technology promotion specialist	Popularization of agricultural technology	Corporation	4–6	30 min
13	Guo**	Male	Master	Engineer	Aerospace	Research institution	4–6	1 h
14	Li**	Male	Doctor	University teacher	Emergency management	University	≥ 10	30 min
15	Jin*	Female	Bachelor	Product designer	Product design	Corporation	1–3	30 min
16	Yue*	Female	Master	Data analyst	Financial data	Corporation	4–6	35 min
17	Zheng**	Male	Doctor	R&D staff	Material research and development	Corporation	1–3	1 h
18	Zhang*	Female	Doctor	University teacher	Research on science and technology policy	University	≥ 10	50 min
19	Wang*	Male	Master	Dispatch control	Electrical engineering	Corporation	7–9	30 min
20	Yuan*	Male	Master	Engineer	Aerospace	Corporation	4–6	25 min
21	Luo*	Male	Doctor	Surgeon	General surgery	Hospital	1–3	40 min
22	Ji*	Male	Doctor	Engineer	Aerospace	Research institution	1–3	25 min
23	Wang*	Male	Doctor	Engineer	Aerospace	Corporation	7–9	30 min
24	Hu**	Male	Doctor	University teacher	Bioengineering	University	4–6	35 min
25	Chen**	Male	Master	User researcher	User analysis	Corporation	1–3	30 min
26	Zou**	Male	Doctor	University teacher	Land resource management	University	≥ 10	40 min
27	Zhang**	Female	Doctor	Researcher	Medical equipment R&D	Research institution	1–3	40 min
28	Li**	Male	Master	Programmer	Image algorithm	Corporation	4–6	35 min
29	Li**	Female	Doctor	Assistant researcher	Science and technology governance	Research institution	4–6	1.5 h

* and ** denote the anonymization of respondents’ names.

In addition to the interview data, we also searched for biographies and news reports as secondary data on 15 scientific and technological personnel, including Qian Xuesen, Wang Dazhong, Lei Jun, Zhang Chaoyang, Zhou Jin, and others. After combining this information with the 29 scientific and technological personnel who were directly surveyed, we amassed a comprehensive collection of 44 interviews and documentary materials, providing diverse perspectives on this demographic. These rich data strengthen the reliability and validity of our research and its conclusions. We subsequently encode the text materials. The primary data from 25 scientists and secondary data from 12 scientists are used to establish the initial database, which is then sorted and refined. Through a three-step coding process, we construct the dimensions of innovative competitive behavior among scientific and technological personnel. The primary data from the remaining 4 technicians and the secondary data from 3 technicians are utilized as test data to verify the saturation of the theoretical framework.

### 3.2 Data analysis and model construction

Through interviews, we found that nearly all scientific and technological personnel exhibit clear innovative competitive behavior, indicating that such behavior is prevalent among them. The following paragraph uses grounded theory to define the dimensions of this behavior.

#### 3.2.1 Open coding

Open coding involves assigning conceptual labels to each sentence or excerpt within the raw interview data that requires coding, effectively transforming this information into a conceptual framework. Since the initial concepts are relatively elementary, numerous, and inevitably overlap, a subsequent refinement process must be conducted to integrate related concepts into cohesive groups, facilitating conceptual classification. To undertake this task, we utilized NVivo 11.0 software, a qualitative analysis tool developed by QSR International in Australia. Owing to its robust data coding capabilities and ability to establish theoretical models from unstructured data, NVivo has been widely employed in research across multiple disciplines (Hutchison et al., 2010). We distilled the data into six subcategories, as detailed in Table 2. Owing to space constraints, we select only representative information from each category. This selected sample, while not exhaustive, aims to encapsulate the essential insights that emerge from our analysis.

**TABLE 2:** Examples of open coding.

Excerpts of information	Conceptualization	Categorization
Recently, ChatGPT has become very popular, and many people are eager to learn and quickly write a related paper, fearing they will fall behind.	Taking the lead in delivering results	The timeliness-based competition behavior in innovation results
It’s just a matter of moving the module from one board to another. It’s been thoroughly and rigorously verified online, accelerating testing and time to market.	Fast testing speed	
It is normal for colleagues to compare who has more innovative achievements.	Quantitative advantage in achievements	The quantity-based competition behavior in innovation results
At that time, in a developed provincial organization, many users relied on mobile phones, resulting in a heavy signaling load. The competitor’s product on the STP a-plane could not handle the large volume of traffic, causing the entire a-plane to crash. All traffic was rerouted to Huawei’s system. However, Huawei’s STP independently supported the network for a week, playing a significant role in ensuring the continuity of the organization’s network. The competitor’s equipment failed to recover for a week, primarily due to insufficient product performance. In contrast, our quality proved to be very reliable.	Quality advantage in achievements	The quality-based competition behavior in innovation results
Our technology must be more advanced and efficient, with more precise and durable devices.	Criteria for evaluating the quality of achievements	
With limited organizational resources, progress will inevitably require competition with others. For instance, if only a few devices are available in the laboratory, it becomes a matter of grabbing them when everyone wants to use them.	Competition for equipment resources	Competitive behavior in innovation resource acquisition
When we apply for projects, we will utilize various channels to gather information and monitor the actions of others involved.	Competition in information acquisition	
Nowadays, with such intense competition, everyone is highly competitive. To stay ahead, it is necessary to manage oneself well and dedicate the time others use for rest and play to learning and work.	Competition in investing time for innovation	Competitive behavior in innovation resource allocation
Indeed, malicious competition may involve providing false information, concealing or not reporting design boundaries, design requirements, design interfaces, and so on to gain an unfair advantage.	Competition in the improper utilization of innovative resources	Competitive behavior in innovation resource utilization
At that time, I proposed “Five Transcendences”: transcending the human brain, transcending the human body, transcending history, transcending oneself, and transcending research and development.	Competition in the proper utilization of innovative resources	

Its naming and meaning are as follows: the quantity-based competition behavior of innovation results refers to the competition for the quantity of innovation results, whereas the quality-based competition behavior pertains to the competition for the quality of those results. The more numerous and higher-quality the innovation achievements are, the easier it becomes to win in the innovation competition. Timeliness-based competitive behavior involves competition for the timeliness of innovations. Improving efficiency and shortening time contribute to success in this type of competition. The three constructs reflect the competition among scientific and technological personnel regarding the quantity, quality, and timeliness of innovative achievements. If scientific and technological personnel can generate more, better, and faster innovative outcomes, their chances of winning in the innovation competition will significantly increase.

Competitive behavior in acquiring innovation resources involves how individuals compete to obtain these valuable assets. It occurs when technology professionals vie with one another for essential tools, funding, and other resources necessary for their innovative projects, aiming to gain a competitive edge. Innovation resource allocation refers to the activity or process undertaken by the resource allocation entity, encompassing the strategic distribution of various elements of innovation resources through a defined methodology. Given limited resources, only a reasonable and effective allocation of innovation resources can lead to higher performance. Competitive behavior in innovation resource allocation reflects how individuals compete to distribute these resources. This includes situations where technology professionals invest more time, energy, and innovative resources in their projects while simultaneously showcasing superior allocation skills regarding funds, equipment, and other vital resources. Competitive behavior in innovation resource utilization pertains to the actions taken by individuals, as they compete for the effective use of these resources. There are numerous methods for utilizing innovative resources, which can be broadly delineated into two categories: legitimate and illegitimate. During innovation activities, as technology professionals strive for competitive advantages, they may sometimes resort to more “aggressive” and illegitimate competitive behaviors, such as sabotaging competitors, setting traps, or withholding information. However, more often than not, they engage in rational utilization of their own innovative resources, aiming to increase their competitiveness through the efficient and ethical exploitation of these resources.

#### 3.2.2 Axial coding

When the primary purpose of open coding is to uncover categories, the critical task of axial coding involves refining and expanding upon the main categories. By bridging the gaps between individual categories and revealing their underlying logical connections, axial coding enriches the nature and aspects of these categories, making them more robust and coherent. Our findings indicate that the six categories identified through open coding exhibit a profound intrinsic connection at the conceptual level. After examining the interrelationships and logical sequences among the various categories, we undertook a reclassification process, leading to the identification of two main categories. The two primary categories and their corresponding initial categories are outlined in Table 3.

**TABLE 3:** The main categories formed by axial coding.

Main category	Initial category	Explanation of the connotation of the main category
Competitive behavior In innovation output	The timeliness-based competition behavior of innovation results	The competitive behavior in innovation output refers to the competition individuals undertake with other actors regarding innovation outcomes. This competition often involves the’ quantity, quality, and timeliness of these outcomes.
	The quantity-based competition behavior of innovation results	
	The quality-based competition behavior of innovation results	
Competitive behavior in innovation input	Competitive behavior in innovation resource acquisition	The competitive behavior in innovation input pertains to the rivalry among individuals and other actors in acquiring, allocating, and utilizing innovation resources.
	Competitive behavior in innovation resource allocation	
	Competitive behavior in innovation resource utilization	

#### 3.2.3 Selective coding

After analyzing the two main categories, we discovered the core category “structure of innovative competitive behavior” (Table 4). The competitive behavior in innovation input and the competitive behavior in innovation output are subordinate to innovative competitive behavior. These two dimensions correspond to the input and output stages of innovative competitive behavior, respectively, reflecting the different performances of innovative competitive behavior at various stages. The competitive behavior of innovation investment is the foundation of innovative competitive behavior, directly determining the effectiveness of innovative competition. The competitive behavior of innovation results marks the conclusion of innovative competitive behavior and serves as the basis for assessing whether the innovation competition can be won.

**TABLE 4:** Core category of selective coding.

Core scope	Main category
The structure of innovative competitive behavior	Competitive behavior in innovation output
	Competitive behavior in innovation input

**FIGURE 4:**
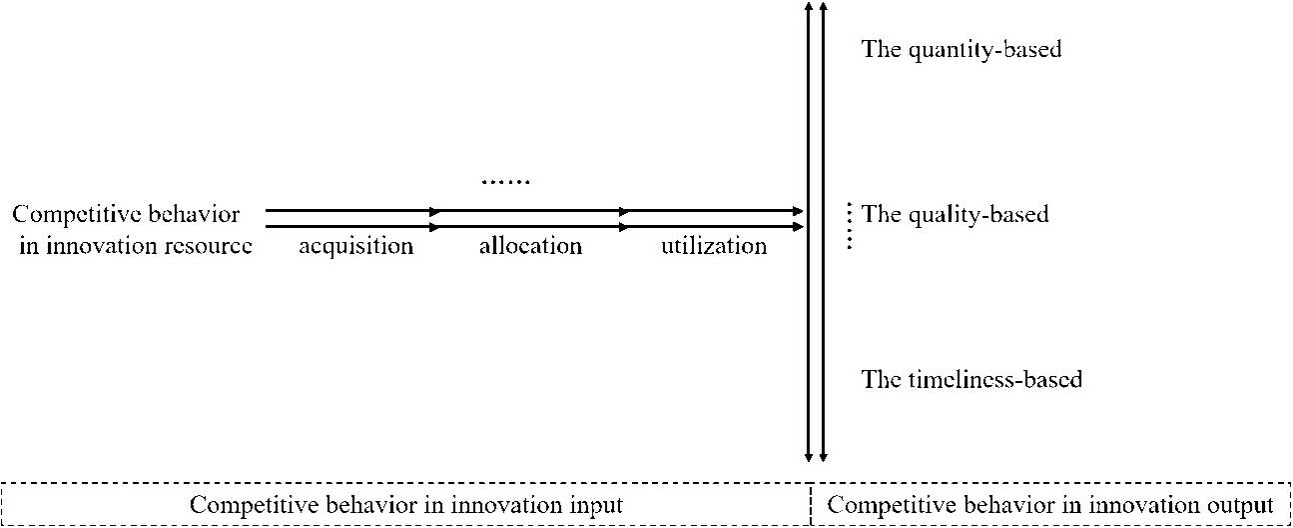
Conceptual components of innovative competitive behavior.

On the basis of the connotation and logical relationship between the core category, the main category, and the initial category, we identify the conceptual components of the innovative competitive behavior of scientific and technological personnel, as shown in Figure 4.

Innovative competitive behavior exists throughout the entire process of innovation competition, including competition in innovation input and competition in innovation outcomes. According to the different stages of innovation resource investment, competitive behavior in innovation input can be divided into competition for the acquisition, allocation, and utilization of innovation resources. The process of competition in innovation input is often unidirectional and irreversible. Once innovation resources are invested and utilized, they cannot be easily withdrawn. Furthermore, the number of actors in competition for innovation input always consists of at least two. Therefore, in Figure 4, two parallel lines with a single arrow and an ellipsis represent the action lines of innovation competition subjects. Competition in innovation outcomes mainly revolves around the quantity, quality, and timing of those outcomes. Since the number of subjects participating in innovation competition activities is always at least two, two parallel lines and an ellipsis in Figure 4 indicate that the innovation outcomes produced by the competition subjects are also at least two.

By aligning the grounded findings with the existing literature, it becomes evident that the categorization of innovation input competitive behavior in this study resonates with the “resource integration process.” Amit and Schoemaker (1993) categorized the resource integration process into resource identification and selection, acquisition, development, and integration; Morgan and Turner (2000) studied resource value creation from four aspects: acquisition, integration, positioning, and maintenance; and Finney et al. (2005) noted that the resource management process includes resource acquisition, integration, market positioning, and maintenance. Although different scholars divide the resource integration process into various stages, the essence of these categorizations reflects the process through which different types of resources are utilized after a series of steps are conducted. From this perspective, the competitive behaviors concerning innovation resource acquisition, allocation, and utilization in this study reflect the process of innovation resource integration. Therefore, on the basis of a comparison with existing research on the resource integration process, it is reasonable to divide innovation input competitive behavior into these three dimensions. This categorization aligns with the academic theories on resource integration and closely matches the practical needs of innovation practices, providing a clear dimensional framework for a deeper understanding of innovation input competitive behavior. From the perspective of innovation achievements, previous research has often emphasized the quantity, quality, and speed of innovation outcomes at the enterprise level (Liu et al., 2021; Kessler and Chakrabarti, 1996), whereas studies on individual innovation outcomes have focused primarily on individual innovation performance (Jiang et al., 2023; Rangus and Černe, 2019), with inadequate attention given to the quantity, quality, and timeliness of individual innovation outcomes. Through grounded theory, this study finds that scientific and technological personnel compete with other actors in terms of the quantity, quality, and speed of innovation outcomes.

**FIGURE 5:**
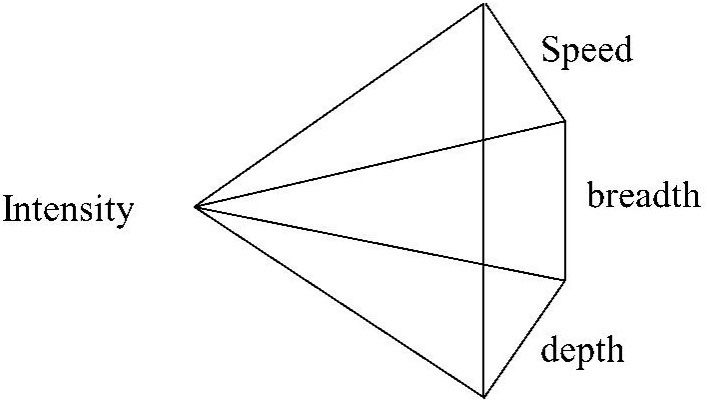
Four-Degree Diamond Model of innovative competitive behavior.

On the basis of the conceptual model of innovative competitive behavior, this study constructs and develops a “Four-Degree Diamond” Model of innovative competitive behavior, as shown in Figure 5. In the competitive behavior of innovation results, quantitative indices serve as extension indices that reflect the breadth of phenomena, whereas qualitative indices function as connotation indices that capture the depth of these phenomena. Therefore, quantity-based competition in innovation results illustrates the breadth of innovation competition, whereas quality-based competition in innovation results represents its depth. Speed relates to competition over time, and timeliness-based competition in innovation results indicates the speed of innovation competition. The depth, breadth, and speed collectively reflect the objective attributes of the innovation results, enriching the connotation of competitive behavior in innovation outcomes. In contrast, competitive behavior related to innovation input encompasses the acquisition, allocation, and utilization of innovation resources, classified according to the sequence of the input process. The competition for innovation inputs primarily signifies the intensity of innovation competition, whether through acquisition, allocation, or utilization of resources. The term “degree” is utilized to encapsulate the different facets of competition in both innovative input and output, as “degree” refers to the level or extent. Across various aspects of competition in innovation input and output, individuals differ in their level or extent of achievement, thus creating competitive possibilities.

The Four-Degree Diamond Model is interconnected with the intensity of innovation investment competition and culminates in the speed, breadth, and depth of innovation performance competition. Intensity, speed, breadth, and depth are intricately intertwined, forming the “Four-Degree Diamond” Model of innovative competitive behavior. Numerous studies have shown a significant positive correlation between investment and performance (Hinloopen, 2003; Zhao et al., 2019; Gu et al., 2016). Accordingly, the intensity of innovation investment competition profoundly influences the success of innovation competition. However, it is important to note that while the intensity of innovation investment competition may influence innovation competition performance, it does not necessarily impact the speed, depth, or breadth of innovation output competition. The intrinsic nature of the competition between innovation investment and innovation achievement dictates this. Individuals may compete with external actors regarding innovation investment, but this does not invariably establish a competitive relationship in terms of innovation performance output. The intensity, speed, breadth, and depth of the Four-Degree Diamond Model are juxtaposed rather than indicative of causal relationships. This model originates from the individual level of technological personnel but also possesses explanatory power at the macro and meso levels. For example, since the commercialization of 5G in 2019, China’s basic telecommunications industry has invested nearly 600 billion yuan in the construction of 5G networks. China’s 5G network construction has rapidly advanced, resulting in the establishment of the world’s largest and most sophisticated 5G network, encompassing all prefecture-level cities and county-level urban areas nationwide (Wang and Liu, 2023). China’s leadership in the intensity, speed, breadth, and depth of 5G network construction surpasses that of other countries, demonstrating the success of China’s innovative competition in 5G network construction and development.

The Four-Degree Diamond Model introduced in this paper represents a profound refinement and expansion of the concept and dimensions of innovative competitive behavior. Technological professionals engaging in vigorous competition during innovative endeavors intensify their pursuit of innovation inputs. Similarly, the same applies to speed, depth, and breadth. Therefore, the Four-Degree Diamond Model, which encapsulates the intensity of competition in innovative inputs as well as the speed, depth, and breadth of competition in innovative outcomes, offers a more precise and vivid portrayal of the extent to which individuals engage in innovative competitive activities. This, in turn, enhances our comprehension of the essence of innovative competitive behavior. By analyzing an individual’s Four-Degree Diamond Model, we can distinguish whether a technological professional is inclined to actively engage in innovative competition or prefers a less competitive stance with a greater focus on personal pursuits. Such insights are valuable for governments, enterprises, and other organizations seeking to understand the innovation competitiveness landscape among their technological personnel. They can utilize this information to fine-tune their management strategies: moderating incentives during intense innovative competition and bolstering them when competition is more tempered. Furthermore, the model serves as an essential reference for management practices in organizations, enabling governments and businesses to allocate resources and tailor interventions on the basis of the unique competitive profiles of their technological workforce.

#### 3.2.4 Saturation test

After we preliminarily determine the structure of innovative competitive behavior, we use the remaining data to conduct a theoretical saturation test. We found no new categories and did not alter the structure of innovative competitive behavior, thus confirming that the theoretical saturation of this study is satisfactory.

## 4 Hypotheses

Drawing from the dimensions of innovative competitive behavior of scientific and technological personnel developed through grounded theory, relevant research hypotheses are proposed and supported by the existing literature.

Innovation activities possess distinct stage characteristics. By utilizing grounded theory to construct dimensions of innovative competitive behavior, this study categorizes the behavior into competitive behavior in innovation input and competitive behavior in innovation output. Scientific and technological personnel compete with other actors in innovation input activities, such as investing more time, effort, and resources; they also compete with others in producing innovation outcomes. Previous research on individual innovation outcomes has focused primarily on individual innovation performance (Jiang et al., 2023; Rangus and Černe, 2019), whereas studies on individual innovation input has often centered on individual innovation initiative and individual innovation effort (Frohman, 1999; Bandura, 1997). Some scholars have highlighted the necessity of promoting individual innovation input to stimulate breakthrough innovation behavior (Fernández-Sastre and Martin-Mayoral, 2017). However, the existing research seldom considers both individual innovation resource input and innovation outcome production simultaneously, even though these two aspects are integral to the entire innovation activity. Innovation input serves as a prerequisite for generating innovation outcomes, offering the necessary resource support for their production, and a close connection exists between the two. Compared with a single-dimensional view of innovation competitive behavior, dividing it into two dimensions—innovation input competitive behavior and innovation outcome competitive behavior—improves our understanding of innovation competitive behavior. Therefore, the following hypotheses are proposed:

*H1*: The concept of innovative competitive behavior comprises two factors, competitive behavior in innovation input and competitive behavior in innovation output, which are positively correlated with each other.

*H2*: The overall fit of the two-factor model, which includes competitive behavior in innovation input and competitive behavior in innovation output, is better than that of the single-factor model of innovation competitive behavior.

## 5 Scale development—quantitative research

In this section, we develop an innovative competitive behavior scale based on the process of “analyzing the initial data → developing the initial scale → pretesting → formal testing” and conduct multiple tests on it.

### 5.1 Initial scale development

The initial materials for designing the Innovative Competitive Behavior Scale consist of the aforementioned interviews with 44 scientific and technological personnel, relevant literature, and existing scales for innovative and competitive behavior. By analyzing these materials, this paper develops an initial scale of innovative competitive behavior from September to December 2023. The specific development process is as follows:

First, grounded theory is used to establish the two main categories of innovative competitive behavior as the foundational framework for scale design. Six initial categories form the core content of these two main categories, and by integrating the relevant scales of innovative and competitive behavior from existing research, the initial questionnaire items are developed. As mentioned earlier, innovative competitive behavior comprises two major dimensions: competition in innovation input and innovation outcomes. Competition in innovation input includes competition for acquiring, allocating, and utilizing innovation resources. In contrast, competition in innovation outcomes covers elements of competition regarding the quantity, quality, and timing of innovation outcomes. On the basis of these findings, this study’s innovative competitive behavior scale consists of two parts: competition in innovation input and innovation outcomes. The items for competition in innovation input comprise three components: competition for the acquisition, allocation, and utilization of innovation resources. The items for competition in innovation outcomes also consist of three components: competition concerning the quantity, quality, and timing of innovation outcomes. Second, the initial questionnaire items align with the interview materials from scientific and technological personnel, and the language used in these materials is analyzed to refine the questionnaire items, resulting in an item pool containing 31 items. Two professors of management and six doctoral students in management were subsequently invited to review each item individually to reduce and revise the expressions with redundant meanings or that do not conform to practical usage, ensuring clear, concise, and easy-to-understand language. For example, the item “I will take measures to produce papers, plans, products, and other innovative outcomes at a faster speed and in a shorter time than others” was modified to “I strive to produce innovative outcomes at a faster speed and in a shorter time than others.” The dimensionally ambiguous item “I actively apply for patents to prevent infringement and theft of innovative outcomes” was removed. Some items that were difficult to evaluate and had unclear expressions, such as “I use knowledge, funds, technology, connections, and other innovation resources more efficiently than others, with less waste” and “Compared with others, I can discard useless equipment, relationships, and other innovation resources in a timely manner,” were also removed. The revised scale was then reviewed by two additional professors of management and five doctoral students in management. The results of this review were consistent with those from the previous round, and no items were altered. Finally, an initial scale for innovative competitive behavior consisting of 14 items was established.

In this part, we sought the guidance of four professors, each an authority in their respective domains of science and technology policy studies, technology transfer and science & technology management, innovation management research, and competition policy research. With over a decade of professional experience in their fields, these scholars have acquired profound expertise by pursuing innovation and competition research. They played a pivotal role in developing rigorous screenings and refining the research scale employed in this study. Furthermore, we collaborated with 11 doctoral candidates in management, whose research interests encompass diverse areas, such as science and technology management, technological innovation management, innovation risk management, competitive intelligence, artificial intelligence research, innovation cooperation and competition, innovation human resource management, and industrial policy studies. These doctoral candidates, who are actively engaged at the forefront of scientific research and striving at the cutting edge of inquiry, provided valuable insights that significantly enhanced the quality of the scale developed in this paper. Importantly, owing to concerns regarding respondent burden and practical constraints, the items incorporated within the scale are inherently limited in their ability to comprehensively capture the full spectrum of phenomena encompassed by the concept of innovative competitive behavior. Consequently, we meticulously designed and selected items grounded in the multiple dimensions of innovative competitive behavior to ensure that each item is closely tied to its core meaning. Our primary objective is to create a scale that efficiently measures innovative competitive behavior while preserving the questionnaire’s brevity and effectiveness. Moreover, prior research has validated the efficacy of concise scales, exemplified by the 6-item (Scott and Bruce, 1994), 9-item (Janssen, 2000), and 13-item (Zhou and George, 2001) scales for innovative behavior, alongside the 3-item scale (Hays and Bendersky, 2015) for competitive behavior, in accurately measuring their respective constructs despite their relatively small number of items. This precedent underscores the feasibility of utilizing a select number of items to achieve reliable measurement. Therefore, the selection of 14 items for our scale is deemed appropriate and in line with established practices in the field.

### 5.2 Preliminary research

The preliminary research for this study was conducted in January 2024. The subjects were scientific and technological personnel from organizations such as science and technology enterprises, research institutes, and universities. The survey questionnaire employed a five-point Likert scale, with scores ranging from 1 to 5 indicating “completely disagree” to “completely agree.” The questionnaires were distributed through online survey platforms and onsite methods. Two hundred questionnaires were distributed during the preliminary research period, and 180 valid questionnaires were collected, resulting in an effective recovery rate of 90%. The analysis was conducted using SPSS 26.0, revealing that this scale has high overall reliability (Cronbach’s α value is 0.908). The purification of indicators was performed using the corrected item-total correlation coefficient (CITC), leading to the deletion of the item “I have more channels for obtaining innovative resources than others” because the CITC value is less than 0.4. The mean value of this item in the preliminary research data is 2.37, indicating that most scientific and technological personnel believe that there are limited channels for obtaining innovative resources, showing a strong contrast compared with other items measuring innovative competitive behavior. The results of the exploratory factor analysis indicated that the KMO value of the scale is 0.862 and that Bartlett’s test of sphericity is significant (p < 0.001), demonstrating that the scale is suitable for factor analysis. Finally, a 2-factor model containing 13 items was derived, which included a factor of competitive behavior in innovation input (F1, 7 items) and a factor of competitive behavior in innovation outcomes (F2, 6 items). This model reveals that the Innovation Input Competitive Behavior Scale comprises three items on the acquisition of innovative resources, two items on the allocation of innovative resources, and two items on the utilization of innovative resources. The Innovation Outcome Competitive Behavior Scale consists of two items each for the quantity, quality, and timing of innovation outcomes. Consistent with the expected structure, the cumulative proportion of variance explained by these two factors reaches 62.505%. The results of the exploratory factor analysis further reinforce the structural dimensions of innovative competitive behavior identified in the qualitative research stage.

### 5.3 Formal research

#### 5.3.1 Data collection and sample overview

The formal survey for this study was conducted from February to April 2024. The scale and data for this study are available for replication, and consent was obtained from all participants. The formal survey objects and the questionnaire distribution method are identical to those used in the presurvey. We distributed 650 questionnaires and received 563 valid responses, resulting in an effective recovery rate of 86.62%. The data are randomly divided into two groups: Group A and Group B. The data in Group A (N = 281) are used for exploratory factor analysis of the structure of innovative competitive behavior, whereas the data in Group B (N = 282) are employed for confirmatory factor analysis. Independent samples t-tests conducted on the two data groups revealed no significant differences in the variables of sex, age, nature of the organization, and highest academic degree currently obtained (p-values are greater than 0.10). According to the United Nations Educational, Scientific and Cultural Organization (UNESCO), “support personnel” in the field of science and technology include personnel such as clerks, secretaries, and administrative managers who are directly involved in work related to science and technology activities (UNESCO, 1979). Therefore, in this survey, the staff engaged in science and technology management and services within the science and technology departments of party and government agencies, along with those who provide technological support to residents and train them in the use of relevant technological tools within neighborhood committees or village committees, are also included in the research scope. The descriptive statistics of the samples used in this study are shown in Table 5.

**TABLE 5:** Sample statistics.

Distributional characteristics		Group a (n = 281)		Group b (n = 282)	
		Sample size	Percentage	Sample size	Percentage
Gender	Male	143	50.89%	154	54.61%
	Female	138	49.11%	128	45.39%
Age	≤ 30	72	25.62%	81	28.72%
	31–45	133	47.33%	140	49.65%
	46–60	62	22.06%	53	18.79%
	≥ 61	14	4.98%	8	2.84%
The highest academic degree currently obtained	Bachelor	115	40.93%	125	44.33%
	Master	107	38.08%	95	33.69%
	Doctor	54	19.22%	55	19.50%
	Others	5	1.78%	7	2.48%
The type of your organization is	Party and government office	35	12.46%	36	12.77%
	Public institution or state-owned enterprise	88	31.32%	77	27.30%
	Enterprise	84	29.89%	98	34.75%
	Social group or neighborhood/village committee	7	2.49%	10	3.55%
	Self-employment	19	6.76%	15	5.32%
	Unemployed	48	17.08%	46	16.31%

In addition, this paper also collected data for each measurement item regarding the innovative competitive behavior of science and technology personnel. The descriptive statistics for the variables are shown in Table 6.

**TABLE 6:** Variable statistics.

Items	Group a (n = 281)		Group b (n = 282)	
	Mean value	Standard deviation	Mean value	Standard deviation
Q1	3.48	1.025	3.79	0.871
Q2	3.66	0.981	3.81	0.843
Q3	3.55	1.072	3.82	0.846
Q4	3.38	1.018	3.73	0.807
Q5	3.46	1.038	3.63	0.764
Q6	3.26	1.062	3.68	0.883
Q7	3.27	1.065	3.59	0.886
Q8	3.42	1.157	3.79	0.919
Q9	3.11	1.212	3.78	0.909
Q10	3.39	1.166	3.8	0.897
Q11	3.46	1.158	3.77	0.955
Q12	3.43	1.141	3.56	0.968
Q13	3.29	1.121	3.63	0.979

#### 5.3.2 Scale analysis and validation

##### 5.3.2.1 Exploratory factor analysis

**FIGURE 6:**
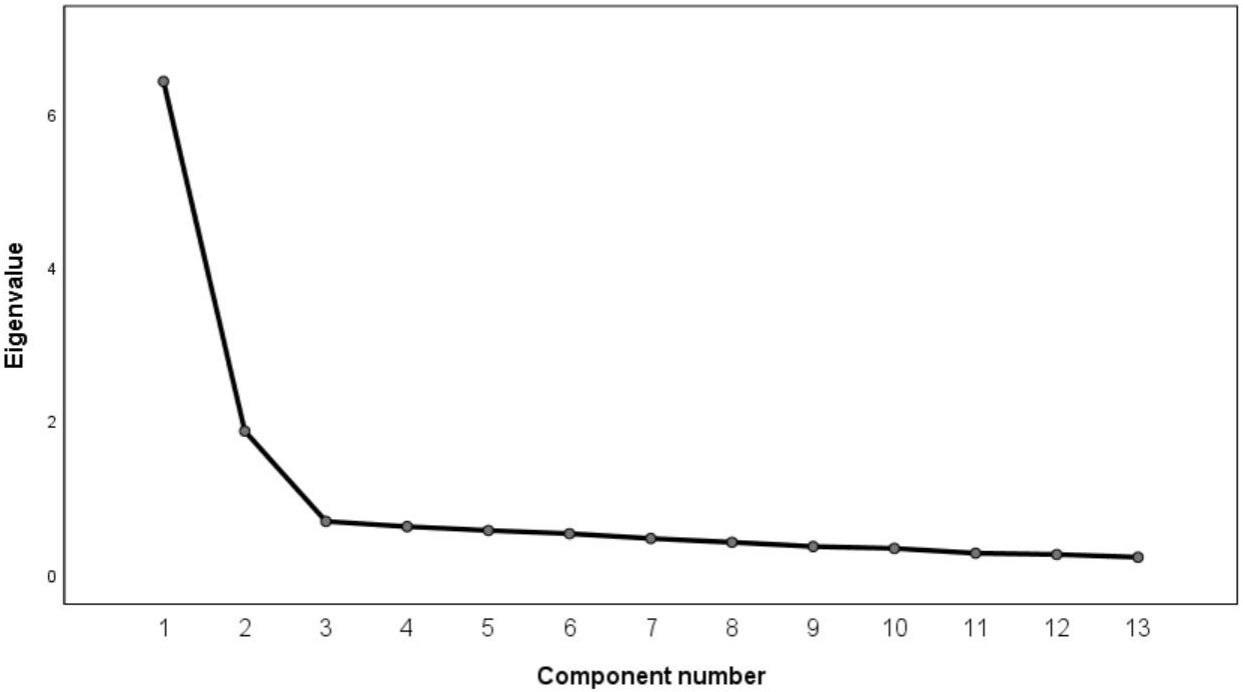
The scree plot.

Group A demonstrated high overall reliability, with a Cronbach’s α value of 0.913. Upon examination, all the corrected item–total correlation (CITC) values exceeded the threshold of 0.4. The exploratory factor analysis revealed that the Kaiser–Meyer–Olkin (KMO) value is 0.917 and that Bartlett’s test of sphericity is significant (p < 0.001), indicating the scale’s suitability for factor analysis. By applying the principal component analysis method and rotating the factors using the maximum varimax method, factors with eigenvalues greater than one are extracted, as shown in Figure 6 and Table 7. Figure 6 shows the scree plot exhibits a steep slope from the first to the second factor, followed by a noticeable inflection point at the third factor. The subsequent indicator variables gradually form a smoother curve, suggesting that selecting two factors is appropriate. The formula for exploratory factor analysis is as follows:


X=Λ⁢F+δ

**TABLE 7:** Results of exploratory factor analysis.

Items	The factor of competitive behavior in innovation input	The factor of competitive behavior in innovation output
Q1 i decide whether to acquire additional resources based on observing others’ acquisitions of innovation resources, such as technology, equipment, and funding.	0.810	
Q2 i showcase my advantages in technology and solutions to the organizers when applying for scientific projects or participating in innovation competitions.	0.781	
Q3 i will compete with others if there are opportunities to obtain beneficial resources like scientific projects, funding, and connections.	0.711	
Q4 i endeavor to devote more study time, energy, and money than others to innovation activities. Q5 my advantage in innovation activities lies in the scientific and rational allocation of time, funds, and equipment.	0.658 0.783	
Q6 when disclosing research protocols and data that may impact stakeholders, i strive to avoid full disclosure or to release simplified versions of the content.	0.738	
Q7 i often learn new research methods and read cutting-edge literature to surpass my peers in innovative competitive activities.	0.751	
Q8 i compete with others regarding the quantity of innovation achievements, such as technological advancements, published papers, and proposed solutions.		0.729
Q9 i strive to produce more innovative achievements, such as papers, patents, and products, to enhance my competitiveness.		0.724
Q10 i compete with others to see who has achieved higher quality innovations, such as publishing in higher-level journals or developing more advanced technologies.		0.836
Q11 i strive to produce high-quality innovation achievements, such as top-tier papers and advanced technical solutions, to stand out.		0.778
Q12 i strive to produce innovative achievements in a shorter time than others.		0.829
Q13 i will announce my innovative achievements to the public as soon as possible to seize the opportunity and prevent others from taking the lead.		0.752
Cronbach’s alpha coefficient	0.894	0.894
Cumulative proportion explained by variance	32.780%	63.624%

In the formula, X represents the observable variables, F denotes the common factors, the factor loadings indicate the items’ relationship to the corresponding factors, and the error term is included.

A two-factor model was obtained in line with the preliminary research results, explaining a cumulative variance of 63.624% (Table 7).

Table 7 shows that innovative competitive behavior consists of two factors: competitive behavior in innovation input and competitive behavior in innovation output. Items related to competitive behavior in acquiring, allocating, and utilizing innovation resources fall under competitive behavior in innovation input. In contrast, items concerning the timeliness, quantity, and quality of competitive behavior regarding innovation results pertain to competitive behavior in innovation output.

##### 5.3.2.2 Confirmatory factor analysis

The mathematical expressions of exploratory factor analysis (EFA) and confirmatory factor analysis (CFA) are identical, but significant differences exist. EFA is a data-driven exploratory tool that identifies the underlying factor structure. In contrast, CFA is a theory-driven validation tool used to verify whether these factor structures align with theoretical assumptions. By combining both methods, the scale’s factor structure is well supported by the data and meets theoretical requirements, thereby enhancing the quality of the scale.

This study employs a competitive model comparison method to conduct confirmatory factor analysis on Group B data, further verifying the appropriateness of the factor structure model and determining whether the two-factor model is optimal. We combine competitive behavior in innovation input and output into a single-factor model, designated as Model 1. We separate competitive behavior in innovation input and innovation achievement into a two-factor model, designated as Model 2. Using Amos 28.0, we perform a comparative analysis of the competitive models and determine the optimal model by comparing fit indices. The model fit data are presented in Table 8. Confirmatory factor analysis evaluates model fit through fit indices, including the chi-square to degrees of freedom ratio (χ^2^/df, with values between 1 and 3 being ideal), the root mean square error of approximation (RMSEA, where less than 0.05 is ideal), and the incremental fit indices (CFI, TLI, and IFI, with values greater than 0.9 being ideal). Table 8 shows that Model 2 fits better than Model 1. Research Hypothesis H2 is valid.

**TABLE 8:** Fit of competitive models.

Model	χ ^2^	Df	χ ^2^/df	TLI	IFI	CFI	RMSEA
Model 1	509.577	65	7.840	0.740	0.785	0.783	0.156
Model 2	171.486	64	2.679	0.936	0.948	0.948	0.077

**FIGURE 7:**
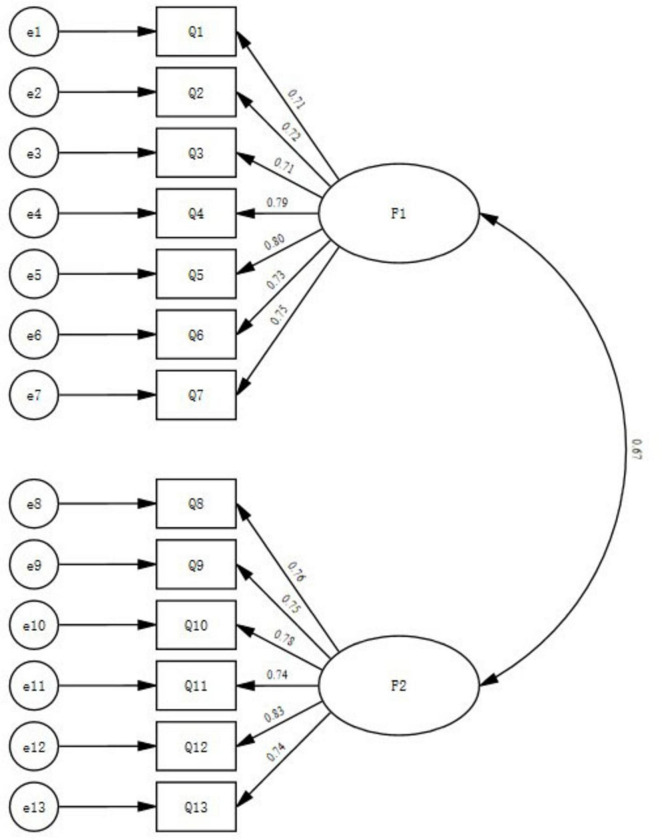
Path diagram of the two-factor structure model of technological personnel’s innovative competitive behavior.

Model 2’s factor loadings for each item surpass 0.50 (Figure 7; Table 9), confirming the optimal two-factor structure model of innovative competitive behavior. The correlation coefficient between the two is relatively high (0.67), therefore, research Hypotheses H1 is valid.

**TABLE 9:** Significance test of path coefficients.

Path			Estimate	S.E.	C.R.	*P*	Factor loading
F1	→	Q1	1	–	–	–	0.705
F1	→	Q2	0.989	0.087	11.332	***	0.721
F1	→	Q3	0.978	0.088	11.161	***	0.710
F1	→	Q4	1.038	0.084	12.36	***	0.790
F1	→	Q5	1.001	0.08	12.582	***	0.805
F1	→	Q6	1.057	0.092	11.543	***	0.735
F1	→	Q7	1.083	0.092	11.788	***	0.751
F2	→	Q8	1	–	–	–	0.756
F2	→	Q9	0.985	0.078	12.706	***	0.752
F2	→	Q10	1.006	0.076	13.195	***	0.778
F2	→	Q11	1.016	0.082	12.466	***	0.739
F2	→	Q12	1.152	0.082	14.087	***	0.826
F2	→	Q13	1.044	0.084	12.491	***	0.741

***p < 0.01.

Furthermore, in Confirmatory Factor Analysis (CFA), it is generally necessary to conduct significance testing on model parameters (such as factor loadings). The core statistic for this test is the Critical Ratio (C.R.), functioning equivalently to a *t*-test. When the C.R. exceeds 3.29, the corresponding significance *p*-value is less than 0.001, indicating that the path coefficient is significant at the 0.001 level and thus strongly supported by the data (denoted by “***” in this study). In the current research, the C.R. values for all items ranged between 11.161 and 14.087 (see Table 9), substantially exceeding the critical value of 3.29, demonstrating that all parameter estimates have achieved a high degree of statistical significance.

Common method bias refers to the artificial covariance between explanatory variables and outcome variables caused by the same data source, measurement environment, survey characteristics, and other factors. This study employed the “Controlling for a Single Unmeasured Latent Method Factor” technique to test for it. Specifically, a common method latent factor was added to the confirmatory factor analysis model, with this factor loading on all measurement items, thereby forming a controlled model. The fit indices of the controlled model were then compared with those of the original model. The results showed that the changes in the main fit indices were as follows: ΔCFI = 0.026, ΔTLI = 0.024, ΔIFI = 0.026, ΔRMSEA = 0.016. All change values were below the threshold of 0.03, indicating that there is no severe common method bias in this study.

##### 5.3.2.3 Reliability test

This paper employs Cronbach’s α coefficient to assess the reliability of all valid questionnaires. The formula for calculating Cronbach’s alpha coefficient is as follows:


$$\alpha =\left(\frac{k}{k-1}\right)\,\left(1-\frac{\Sigma_{i=1}^{k}\,S_{i}^{2}}{S_{T}^{2}}\right)$$

In the formula, k is the number of items in the scale; $S_{i}^{2}$ is the variance of the responses to the i-th item across all respondents; and $S_{T}^{2}$ is the variance of the total scores across all respondents. The results indicate that the reliability coefficients for competitive behavior in innovation input and competitive behavior in innovation output are 0.899 and 0.897, respectively, both exceeding 0.7. The total scale reliability coefficient is 0.919. Therefore, this study’s innovative competitive behavior scale has high reliability.

##### 5.3.2.4 Validity test

We conducted content validity and construct validity tests to assess the validity of the innovative competitive behavior scale. The scale’s items were developed using interview records and relevant literature. Management experts and researchers were invited to perform repeated analyses, classifications, and reviews of the scale items. In addition, we removed inappropriate items using quantitative analysis methods. This rigorous process ensures the appropriateness of item classification and the accuracy of item descriptions, indicating a high level of content validity for the scale.

Structural validity includes both convergent validity and discriminant validity. Convergent validity focuses on whether items measuring the same underlying trait can cluster together on the same factor. This study uses standardized factor loadings, average variance extracted (AVE), and composite reliability (CR) to evaluate the convergent validity of the scale. Generally, convergent validity is considered adequate when the AVE value exceeds 0.5 and the CR value surpasses 0.7. The formulas are as follows:


$$A\,V\,E=\frac{\Sigma \,\lambda_{i}^{2}}{\Sigma \,\lambda_{i}^{2}+\Sigma \,\theta_{i}}$$

$$C\,R=\frac{{\left(\Sigma \,\lambda_{i}\right)}^{2}}{{\left(\Sigma \,\lambda_{i}\right)}^{2}+\Sigma \,\theta_{i}}$$

In the formula, λi represents the standardized factor loading of the i-th item on the latent variable, and θi represents the error variance of the i-th item. The standardized factor loadings for the entire sample are all greater than 0.5. As shown in Table 10, all CR values are greater than 0.7. Consequently, the scale in this paper demonstrates good convergent validity. Regarding discriminant validity, the square root of the AVE values of the two latent variables exceeds the correlation coefficient, which aligns with the criteria established by Fornell and Larcker (1981). This indicates that the scale exhibits high discriminant validity.

**TABLE 10:** The two-factor correlation coefficient of innovative competitive behavior.

Variable	AVE	CR	Correlation coefficient	The square root of AVE
Competitive behavior in innovation input	0.563	0.900	0.652***	0.750
Competitive behavior in innovation output	0.597	0.899		0.773

***p < 0.01.

## 6 Discussion

### 6.1 Further analysis of the concept of innovative competitive behavior

With respect to the concept of “innovative competitive behavior,” the following discussion is provided. First, innovative competitive behavior must involve both innovation and competition. It requires competitive behavior in innovation activities and innovative behavior in competitive activities; otherwise, it is not considered innovative competitive behavior. Second, in constructing the dimensions of innovative competitive behavior using grounded theory, this study divides it into competitive behavior in innovation input and competitive behavior in innovation achievement from a process perspective. This includes aspects such as keeping innovation information confidential, seizing innovation resources, and being the first to produce innovative outcomes, all of which reflect innovative competitive behaviors. Innovative competitive behavior can be categorized into different types on the basis of varying criteria. For example, it can be classified as active or passive, long-term or short-term, intense or mild, routine or emergency, and overt or covert competitive innovative behavior, among others. This study emphasizes innovative competitive behavior from a process perspective to highlight what subjects are competing for at different innovation stages, essentially answering “what is being competed for.” Other classifications, such as active or passive, long-term or short-term, intense or mild, only partially reflect facets of innovative competitive behavior and do not adequately underscore the competitive content within innovation activities. Thus, the connotation of innovative competition indicated by these other criteria is relatively limited. Third, innovative competitive behavior has certain connections and distinctions from deviant, pseudo-innovation, and disruptive technological innovation behavior. Deviant innovation behavior refers to employees who firmly believe that their efforts enhance the organization’s innovative returns, even when such actions violate managerial directives to halt new ideas. They persist in realizing their innovative concepts (Mainemelis, 2010), emphasizing confrontation and deviation. The similarity between innovative competitive behavior and deviant innovation behavior lies in their confrontational characteristics, and deviant innovation behavior is significant for competing for innovative ideas. However, innovative competitive behavior does not always occur when managerial orders are disregarded. Thus, the context of innovative competitive behavior lacks a precise definition. Pseudo-innovation appears to align with the goals of the socioeconomic system or subsystem; however, it negatively impacts it over time, diminishing the system’s efficiency (Haustein and Maier, 1980). Although it is referred to as innovation, it does not result in substantial improvements. When pseudo-innovation aims to achieve a short-term competitive advantage, it may be regarded as innovative competitive behavior. Conversely, if the goal of pseudo-innovation is not competition but merely to fulfill work requirements or please superiors, it does not fall under innovative competitive behavior. Disruptive innovation emerges from the nonmainstream low-end market and disrupts the competitive advantage of established enterprises (Christensen, 1997). The low-end and niche markets break existing competitive rules, create new markets and value networks, and offer innovative opportunities for small and medium-sized latecomer enterprises (Chen et al., 2019). Disruptive technological innovation behavior refers to actions taken by enterprises, individuals, etc., in disruptive innovation aimed at altering existing market or industry structures by introducing disruptive new technologies. This may include developing breakthrough technologies and adopting completely new business models. Essentially, disruptive technological innovation behavior seeks to overturn traditional technologies and markets, embodying innovative competition; it represents competitive behavior against traditional technology and markets in innovation activities while simultaneously serving as a strategy for gaining advantages through innovation in competitive endeavors. Both disruptive technological and innovative competitive behavior achieve competitive advantages through innovation, but they also differ. With respect to application scope, disruptive technological innovation behavior specifically targets innovative activities capable of subverting existing market or technological patterns. In contrast, innovative competitive behavior encompasses a broader concept involving disruptive technology and extending to cutting-edge technology, scientific research, achievement transformation, and various aspects of life. Therefore, while disruptive technological innovation behavior that alters competitive patterns through innovation can be viewed as a type of innovative competitive behavior, not all innovative competitive behaviors yield disruptive innovation.

### 6.2 Theoretical and practical implications

The proposal of the “innovative competitive behavior” concept, the construction of its dimensions, the development of the “Four-Degree Diamond” model, and the formulation of the scale represent a generalization and refinement of a phenomenon and serve as a systematic and universal theoretical distillation. In contrast to previous research that focused primarily on innovative competition at the organizational level, this study pioneers a new frontier by integrating innovation and competition at the micro-individual level. By delving into individual innovative competition and specifically examining the innovative competitive behavior of scientific and technological personnel as creators of novel knowledge, this work enriches and expands the theoretical frameworks of innovation theory, competition theory, and, ultimately, innovative competition theory. Moreover, innovative competitive behavior originates from social comparison theory in psychology, and our research broadens the practical application scope of social comparison theory.

Innovative competitive behavior serves as a precursor to innovation performance, and a profound understanding of this behavior offers valuable insights for guiding technological innovation management practices. It enables governments and enterprises to effectively manage the innovative competitive dynamics among scientific and technological personnel, motivating and directing their engagement in innovative endeavors. Consequently, this enhances the strategic management of scientific and technological human resources, ultimately facilitating the resolution of critical technological challenges. This approach significantly contributes to bolstering innovation capabilities, increasing innovation performance, and advancing China’s quest for high-level technological self-reliance and self-improvement, thereby leading the nation into a journey of high-quality development. For ordinary individuals, understanding the competitive situation of peers and participating in innovative competitive activities regarding the intensity of innovation input, along with the speed, depth, and breadth of innovation outcomes, can assist with self-management, stimulate personal innovation enthusiasm, enhance competitive innovation capabilities, and improve competitive innovation performance.

### 6.3 Limitations and future research

The current research is not without its limitations. First, a crucial step of external validation was overlooked following the construction of our innovative competitive behavior scale. Second, all measurement instruments employed in this study rely on self-reports, which may introduce biases into the results. Nevertheless, we have tried to mitigate these biases by implementing rigorous quality checks throughout the data collection and analysis. Therefore, the conceptualization of innovative competitive behavior, the delineation of its dimensions, and the development of the scale merely represent the inaugural steps in individual innovation competition research.

Future endeavors must include various external validations of the innovative competitive behavior scale to strengthen the reliability of our conclusions. Furthermore, scholars should investigate the influencing factors and mechanisms behind innovative competitive behavior. It is crucial to recognize that innovative competitive behavior does not always enhance innovation performance; excessive intensity may, paradoxically, hinder innovation. Thus, attention must be given to the potential consequences of this behavior, ensuring a comprehensive understanding of its implications. Ultimately, translating the study of innovative competitive behavior into practical applications is essential. In light of the various innovative competitive scenarios and the complex nature of scientific and technological personnel, with their diverse categories and individual needs, it is vital to explore how organizations, including governments and enterprises, can effectively manage innovative competitive activities, balance competition and cooperation, and devise strategies to successfully motivate and engage these personnel in driving innovation forward.

## 7 Conclusion

Currently, research on both innovation and competition is extensive, but the field of innovation competition remains underdeveloped. In this area, the academic community has focused more on studying innovation competition at the organizational level, emphasizing firms, governments, and other entities. Furthermore, the research on individual-level innovation competition has focused primarily on innovation contests and academic competition, neglecting individual innovative competitive behavior. At the individual level, numerous studies have undertaken conceptualizations and scale developments for innovative and competitive behavior. However, while social comparison theory is relatively established and the concept of competitive behavior related to innovative competition was introduced earlier, the idea of individual innovative competitive behavior, along with its dimensions and psychological scales, has yet to be established. This study examines the prevalent yet understudied phenomenon of “competition in innovative activities” among individuals, which is widely observed in reality and encouraged by governments. It introduces the concept of “innovative competitive behavior,” defining its scope and boundaries, distinguishing it from deviant, pseudo-innovation, and disruptive technological innovation behavior. We define individual-level innovative competitive behavior as the activities individuals engage in to achieve specific goals while competing with others in the generation, evaluation, execution, facilitation, and promotion of new ideas. The innovative competitive behavior is characterized by selfish, confrontational, and dynamic traits. A grounded theory approach was subsequently adopted to construct the dimensions of innovative competitive behavior. Our research indicates that innovative competitive behavior comprises two dimensions: competitive behavior in innovation input, including competition in acquiring, allocating, and utilizing innovation resources, and competitive behavior in innovation output, involving competition in quantity, quality, and innovation outcomes. Based on this, we propose the Four-Degree Diamond Model and put forward the research hypotheses for the innovative competitive behavior scale. The Four-Degree Diamond Model can showcase the intensity, speed, breadth, and depth of innovative competitive behavior, offering a more vivid depiction of the innovative competitive situation of scientific and technological personnel. The innovative competitive behavior scale covers each dimension of innovative competitive behavior. We conducted hypothesis testing using rigorous quantitative analysis methods, and all hypotheses were confirmed, further proving that the scale we developed has good reliability and validity.

## Data Availability

The original contributions presented in the study are included in the article/Supplementary material, further inquiries can be directed to the corresponding author.
