# Supplementary material for: The concept, dimensions, and measurement of innovative competitive behavior of Chinese scientific and technological personnel
Source: Front Psychol. 2025 Nov 19;16:1449998. doi: 10.3389/fpsyg.2025.1449998 (PMC12672339; doi:10.3389/fpsyg.2025.1449998)
Supplement: Supplementary file 1 [file Supplementary_file_1.docx]

**Appendix I: Interview Outline on Innovative Competitive Behavior of Scientific and Technological Personnel**

Dear Respected Scientific and Technological Personnel：

Thank you for taking time out of your busy schedule to participate in the research project conducted by the School of Public Administration at Beihang University. This interview focuses on the "Innovative Competitive Behavior of Scientific and Technological Personnel," aiming to understand the current competitive phenomena and experiences of Chinese scientific and technical personnel in innovation activities, to explore their understanding and perceptions of innovative competitive behaviors. The interview will be conducted in a semi-structured format, and there are no right or wrong answers. Please respond based on your actual situation. We encourage you to speak freely; your sincere cooperation is essential for the success of this study. We promise to keep your responses strictly confidential, and all information will be used solely for research purposes. We deeply appreciate your support and cooperation in this study. The interview questions are as follows:

Innovative competitive behavior is the activities individuals engage in to achieve specific goals while competing with others in the generation, evaluation, execution, facilitation, and promotion of new ideas.

1. Have you or any of your colleagues or friends ever engaged in innovative competitive behaviors? Could you select a few memorable examples and describe them in detail?

2. Do you frequently engage in innovative competitive behaviors?

3.Under what circumstances would you engage in innovative competitive behaviors?

4.How do you compete with others in innovation activities?

5.When your competitors engage in innovative competitive behaviors, how do you typically respond?

6.What is your evaluation of innovative competitive behaviors? Do you think they are more beneficial or detrimental?

7.Regarding "innovative competitive behaviors," is there anything else you would like to add?
