# Supplementary material for: The concept, dimensions, and measurement of innovative competitive behavior of Chinese scientific and technological personnel
Source: Front Psychol. 2025 Nov 19;16:1449998. doi: 10.3389/fpsyg.2025.1449998 (PMC12672339; doi:10.3389/fpsyg.2025.1449998)
Supplement: Supplementary file 2 [file Supplementary_file_2.docx]

**Appendix II: Measurement Questionnaire of Innovative Competitive Behavior for Scientific and Technological Personnel**

Dear Respected Scientific and Technological Personnel：

Thank you for taking the time out of your busy schedule to participate in the research conducted by the School of Public Administration, Beihang University. This survey aims to understand the actual situation of innovative competitive behaviors adopted by scientific and technological personnel. The survey is conducted anonymously, and there are no right or wrong answers. Please respond based on your actual situation. Your genuine feedback is very important to us! We solemnly promise that the research data will be kept strictly confidential, and all information will only be used for scientific research. We sincerely appreciate your support and cooperation!

**Table II.1 Basic Information Statistics**

| Part One: Basic Information | Option |
| --- | --- |
| (1) Your gender is | 1. Male |
|  | 2. Female |
| (2) Your age is | 1. Under 30 years old  2. 31 - 45 years old  3. 46 - 60 years old  4. 61 years old and above |
| (3) Which province are you from? | Beijing; Tianjin; Hebei; Shanxi; Inner Mongolia;  Shanghai; Jiangsu; Zhejiang; Shandong; Fujian; Anhui;  Liaoning; Jilin; Heilongjiang;  Hubei; Hunan; Henan; Jiangxi;  Guangdong; Guangxi; Hainan;  Sichuan; Chongqing; Guizhou; Yunnan; Tibet;  Shanxi; Gansu; Xinjiang; Qinghai; Ningxia |

The following items describe the situations related to innovative competitive behavior. Please check "√" on the options that you agree with. The scale is as follows: 1 = "Completely disagree"; 2 = "Somewhat disagree"; 3 = "Slightly disagree"; 4 = "Neutral"; 5 = "Slightly agree"; 6 = "Somewhat agree"; 7 = "Completely agree".

**Table II.2 Innovative Competitive Behavior Scale for Scientific and Technological Personnel**

| Item | 1234567 |
| --- | --- |
| Q1 I decide whether to acquire additional resources based on observing others' acquisitions of innovation resources, such as technology, equipment, and funding. |  |
| Q2 I showcase my advantages in technology and solutions to the organizers when applying for scientific projects or participating in innovation competitions. |  |
| Q3 I will compete with others if there are opportunities to obtain beneficial resources like scientific projects, funding, and connections. |  |
| Q4 I endeavor to devote more study time, energy, and money than others to innovation activities. |  |
| Q5 My advantage in innovation activities lies in the scientific and rational allocation of time, funds, and equipment. |  |
| Q6 When disclosing research protocols and data that may impact stakeholders, I strive to avoid full disclosure or to release simplified versions of the content. |  |
| Q7 I often learn new research methods and read cutting-edge literature to surpass my peers in innovative competitive activities. |  |
| Q8 I compete with others regarding the quantity of innovation achievements, such as technological advancements, published papers, and proposed solutions. |  |
| Q9 I strive to produce more innovative achievements, such as papers, patents, and products, to enhance my competitiveness. |  |
| Q10 I compete with others to see who has achieved higher quality innovations, such as publishing in higher-level journals or developing more advanced technologies. |  |
| Q11 I strive to produce high-quality innovation achievements, such as top-tier papers and advanced technical solutions, to stand out. |  |
| Q12 I strive to produce innovative achievements in a shorter time than others. |  |
| Q13 I will announce my innovative achievements to the public as soon as possible to seize the opportunity and prevent others from taking the lead. |  |

The questionnaire has come to an end. Thank you for your support!
